# Carbon Dioxide Hydrogenation by Means of Plasmonic Resonance Activation in Silica Aerogel Media

**DOI:** 10.3390/ma11112134

**Published:** 2018-10-30

**Authors:** Sergio Muñoz, Alexander Navarrete, Ángel Martín, Roland Dittmeyer, M. José Cocero

**Affiliations:** 1 BioEcoUVa, Bioeconomy Research Institute, High Pressure Processes Group, Department of Chemical Engineering and Environmental Technology, University of Valladolid, C/Prado de la Magdalena s/n, 47011 Valladolid, Spain; sergiomp@iq.uva.es (S.M.); mamaan@iq.uva.es (Á.M.); mjcocero@iq.uva.es (M.J.C.); 2 Institute for Micro Process Engineering, Karlsruhe Institute of Technology (KIT), Hermann-von-Helmholtz-Platz 1, 76344 Eggenstein-Leopoldshafen, Germany; roland.dittmeyer@kit.edu

**Keywords:** CO_2_ hydrogenation, photocatalysis, surface plasmon resonance, solar fuels

## Abstract

Surface Plasmon Resonance can be used to activate zinc oxide/copper catalysts in order to perform the carbon dioxide hydrogenation reaction by means of light energy, avoiding high-temperature processes. The synthesis and impregnation methods have been designed to fill glass microreactors with ZnO/Cu nanoparticles supported on transparent silica aerogels to maximize the light absorbed by the catalyst. A LED device surrounding the glass microreactors provided white light to activate the catalyst homogeneously throughout the reactor. Temperature, pressure, amount of catalyst and gases flow were studied as possible variables to enhance the process trying to maximize CO_2_ conversion rates, achieving the best results working at high pressures. The use of transparent SiO_2_ Aerogels as supports for photocatalytic gas phase reactions even under high-pressure conditions is demonstrated.

## 1. Introduction

Carbon dioxide conversion into useful products is attracting much attention in the last years as a way to reduce the greenhouse effect and its dramatic consequences for the planet [1,2]. Among the options to perform this CO_2_ conversion, photocatalytic processes using solar energy represent an interesting way to transform CO_2_ due to the use of a renewable energy. CO_2_ can be transformed into methanol, which is a chemical commodity and can store the energy from the sun [3,4]. Transforming CO_2_ into methanol using solar energy generates, therefore, two benefits at the same time: the reduction of CO_2_ emissions and the storage of energy from renewable sources.

Photocatalytic CO_2_ transformation usually shows low conversions because the efficiency of the process is limited [5]. One of the reasons is related to the low amount of light energy that most of the catalysts absorb in the visible or ultraviolet band. Surface Plasmon Resonance (SPR) is a phenomenon exhibited by some metallic nanostructures, which results in a higher light energy absorbance that can be controlled with the shape and size of the nanoparticles. One area where the SPR effect has found application is in biosensing [6,7]. However, in the last few years, its application has extended to photocatalysis [8], which is the main topic of this paper. Commonly, gold and silver nanoparticles have been studied to enhance SPR effect [9,10], although other metals such as copper also produce this effect [11]. Copper has been widely used for carbon dioxide hydrogenation in industry combined with the use of zinc oxide in order to produce methanol with high selectivity [12]. For this reason, a combination of both materials Cu/ZnO could be also used to perform carbon dioxide hydrogenation by means of solar energy using SPR as a way to enhance the efficiency of the process and to maximize the CO_2_ transformation.

SPR effect depends on copper nanoparticles size, whose variation can modify the absorption wavelength of visual light [13]. Carbon dioxide hydrogenation is performed in the contact area between zinc oxide and copper nanoparticles [14], giving a crucial importance to maximize this contact area with the synthesis method chosen. For this reason, the synthesis method must produce discrete copper nanoparticles whose final size can be controlled, and then these copper nanoparticles must be deposited onto zinc oxide nanorods avoiding agglomeration.

Carbon dioxide and hydrogen can produce methanol directly in a one-step reaction. However, the conversion is increased when the transformation is carried out in two steps [15]. First, carbon dioxide is transformed in carbon monoxide (endothermic), and after this CO again with hydrogen produce methanol (exothermic).

In this work, glass microchannels were selected to perform the carbon dioxide hydrogenation because they provided great homogeneity in the light distribution through the reactor [16]. Glass microreactors avoided light absorption interferences, allowing to study the influence of the material properties and reaction conditions on CO_2_ conversion.

In order to fix the catalyst inside the microchannels, a support was required. This support must provide high surface areas in order to load the bimetallic catalyst and, at the same time, allow that enough light is transmitted to the catalyst to activate it. Silica aerogels are micro-mesoporous materials with high surface areas (400–1500 m^2^/g) and high visible transparency [17], properties that placed them as the best option to be used as a support in this work.

Therefore, a bimetallic catalyst ZnO/Cu supported in silica aerogels can be activated with visual light and be used to perform carbon dioxide hydrogenation in glass microchannels. In a previous work, the viability of the idea was tested with good results in a setup described in [18], opening the possibility to improve the process optimizing the reaction parameters. With the purpose of increasing CO_2_ conversion rate, a complete study of the influence of some reaction variables (temperature, pressure, catalyst amount and reactants proportion) has been performed in this work. With this work, we aim to open new opportunities for the integration of plasmonic photocatalysts in future scalable reaction systems to produce useful compounds as such methanol using solar energy. 

## 2. Materials and Methods 

### 2.1. Reagents

The chemicals used during this stage were: Zinc acetate dihydrate (>98%), oleylamine (70%), tetramethyl orthosilicate (98%), ammonia (30%), triethylene glycol (99%) were purchased from Sigma-Aldrich (St. Louis, MO, USA). Ethylene glycol (99.5%) (Merck, Kenilworth, NJ, USA). Copper acetate monohydrate (99.9%) was purchased from Alfa Aesar (Haverhill, MA, USA). Methanol (99.8%) was purchased from Panreac (Barcelona, Spain).

### 2.2. Synthesis of Plasmonic Catalyst

Following the method proposed by Tan et al. (2013) [19], zinc oxide nanorods were developed firstly, and then copper nanoparticles were deposited onto in a second step, controlling the final size of the nanoparticles and the wavelength where visual light was absorbed.

Oleylamine/zinc acetate ratio was the most important parameter that defined the final shape of zinc oxide nanoparticles. In order to produce nanorods, 3 mmol of zinc acetate were mixed with 1.3 mL of oleylamine in a three-necked flask. After degassing the mixture at 80 °C for 45 min in a vacuum atmosphere, a nitrogen stream was introduced in the system and the temperature was increased to 220 °C for 15 min. After this, the final product obtained was a white paste stuck to the bottom of the flask. 10 mL of ethanol were used with stirring to remove the solid product, and then the mixture was centrifuged (centrifuge Kubota 5100, Tokyo, Japan) at 5000 rpm in order to isolate the precipitate. The solid product was washed again twice with 5 mL of ethanol to ensure a perfect removal of the reactants. ZnO nanorods prepared were redispersed in 20 mL of triethylene glycol for two hours by sonication, and then stirred under room conditions overnight.

After that, a dual glycol system was prepared to deposit copper oxide nanoparticles onto the ZnO nanorods. Copper acetate (1.5 mmol) was added in a second pot to 4 mL of ethylene glycol, using sonication for 1h to create a homogeneous solution. Ethylene glycol (2 mL) was also added to the zinc oxide vessel, and the solution was transferred to a three-necked flask again to be degassed at ambient temperature for 10 min. Then, the copper acetate solution was placed in an addition funnel connected to the three-necked flask with the copper oxide, which was heated at 190 °C under nitrogen purging. With the aim of avoiding agglomeration, copper acetate solution was added slowly drop by drop during 10 min. After this, the solution was cooled, washed and centrifuged three times with isopropanol at 5000 rpm. The mass of the solid obtained was then registered. 

### 2.3. Synthesis and Impregnation of Silica Aerogels

The silica gels were synthesized following the method used by Sanz-Moral et al. (2014) [20]. The precursors used were tetramethyl orthosilicate (TMOS), methanol, water and ammonia in a 1:2.3:3.84:0.012 molar ratio. The amount of ammonia was the controlling parameter for the gelation time. Depending on the catalyst load, which also affected the gelation time, molar ratio TMOS: ammonia was varied from 1:0.012 to 1:0.12 in order to achieve the right gelation time. We adjusted the amount of silica precursor added in accordance to the required catalytic load (7.0%, 10.0%, 13.0%) based on the mass of Cu/ZnO catalyst obtained in the previous step.

Silica aerogels also represented a benefit for subsequent impregnation in the glass microchannels. Aerogels were produced through the sol-gel route, mixing all the precursors in a liquid state under stirring to generate the hydrogel. These precursors included methanol, which was used to suspend the catalyst nanoparticles by sonication. After that, methanol was mixed with the other precursors generating the silica net, while retaining the Cu/ZnO catalyst inside. This silica structure was in a liquid form for a few seconds, which allowed the impregnation in glass microreactors by using just a syringe. Then, the gelation process took place directly inside the microreactors obtaining a hydrogel well attached to the walls of the microchannels and avoiding problems of introducing a solid catalyst in a narrow microchannel. In this way, gelation time became the key factor for the catalyst impregnation inside the microreactors. The injection with the syringe in a liquid state required gelation times not too short. However, if the gelation process were too slow, the catalyst would precipitate and the final structure would not be homogeneous. 

First, methanol with the catalyst nanoparticles was mixed with TMOS in a small glass and stirred for a few minutes. At the same time, a second glass beaker with water and ammonia was also stirred and covered to avoid ammonia evaporation. Then, both solutions were mixed and the syringe was used to fill the microreactor. After a few seconds, the liquid turned into the hydrogel with the catalyst inside showing good adhesion and homogeneity along the entire length of the glass microreactor (15 cm).

### 2.4. Aerogels Supercritical Drying

Before carrying out the drying with supercritical CO_2_, an intermediate step was required for aging the hydrogels [21]. In this stage, the microreactors were placed in a vessel filled with methanol and closed for one week. The aim was to remove the water from the structure producing alcogels, which were dried later with supercritical CO_2_. In order to ensure no water remained in the structure, methanol was renewed twice during the week.

**Figure 1:**
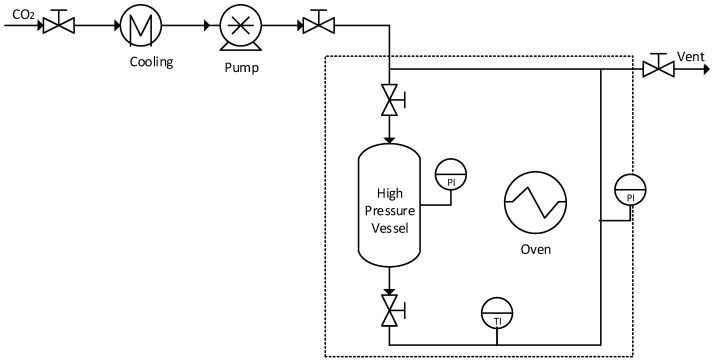
Silica aerogels drying plant.

After that, the alcogels were ready to be dried with supercritical CO_2_. The experimental plant for aerogels drying (Figure 1) had an oven to heat the system at 45 °C, and CO_2_ was pumped at 110 bar above the supercritical point [20]. Inside the oven, there was a high-pressure vessel that was filled with methanol before microchannels were placed inside.

When the high-pressure vessel was closed, the upper valve was slightly opened to introduce the supercritical carbon dioxide and to increase slowly the pressure (if there were a sudden change of pressure inside the vessel, the aerogels would be broken). After the pressure in the vessel reached 110 bar, the lower valve was also opened in order that carbon dioxide started to circulate through the complete system for 45 min. As methanol has not a high solubility in supercritical CO_2_, four drying cycles were performed, renewing CO_2_ between each cycle to ensure a complete drying of methanol. The final products were the aerogels correctly formed and with great adherence to the microchannel walls.

### 2.5. Experimental Reaction Plant

**Figure 2:**
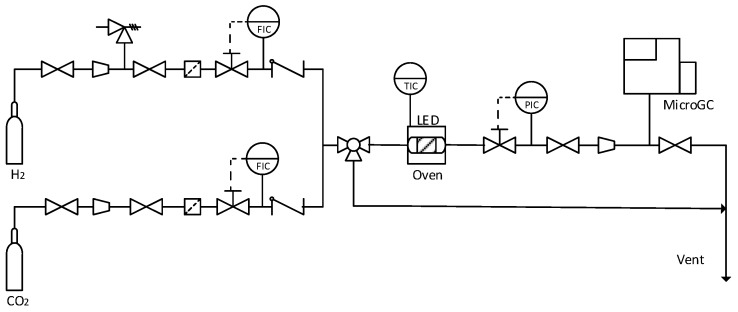
Carbon dioxide hydrogenation experimental plant.

After microreactors were prepared to perform the reaction, they were carried to the experimental setup where carbon dioxide hydrogenation was performed. This plant (Figure 2) was used in previous work to test the reactor concept with good results [18].

The experimental setup was designed to be able to control the reaction parameters that can have an influence on the carbon dioxide transformation, such as pressure, temperature and gas inlet flows. The plant had two flow mass meter/controllers (EL-Flow F-200, Bronkhorst, The Netherlands) for H_2_ and CO_2_ with ranges up to 1 mL/min. The temperature was controlled by placing the microreactors inside a gas chromatography oven (Agilent 7890, Santa Clara, CA, USA) and the pressure was controlled by a pressure meter/controller (EL-Press series, Bronkhorst, The Netherlands) located at the exit of the oven. 

The microreactor consisted of a 0.5 mm ID glass capillary with an external diameter of 5 mm (Schott Duran, Mainz, Germany). The microreactor was surrounded by a set of LEDs (Superbright, inspired LED), which provided a nominal power of 9780 W/m^2^ of white light in the most homogeneous way to illuminate correctly the entire reactor [18]. The gas outlet stream was measured with a Micro Gas Chromatograph (CP-4900, Varian, Palo Alto, CA, USA) equipped with two columns: a poraplot 10 m and a 5A molsieve. 

### 2.6. Catalyst Characterization

Light absorbance tests for the ZnO/Cu catalyst were performed using a UV-Vis Spectrophotometer (Shimadzu UV 2550, Shimadzu, Kyoto, Japan). Transmission Electron Microscopy was carried out with a JEOL JEM-2100F UHR (JEOL, Tokyo, Japan) for catalyst nanoparticles, and with a JEOL JEM-FS2200 HRP (JEOL, Tokyo, Japan) for silica nanocomposites. The chemical structure of the aerogel was studied by Fourier Transform Infrared Spectroscopy (FTIR model TENSOR from Bruker, Billerica, MA, USA). Accelerated Surface Area and Porosimetry Systems (ASAP 2020 and 2420 from Micromeritics, Atlanta, GA, USA) were used to calculate the specific surface area and to determine the nitrogen isothermal adsorption—desorption curve.

## 3. Results and Discussion

### 3.1. Catalyst Characterization

**Figure 3:**
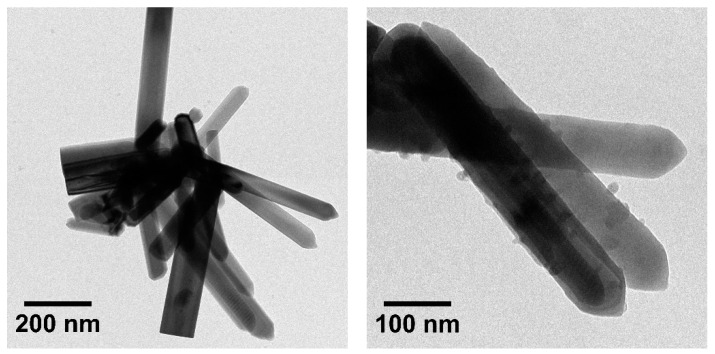
TEM images of ZnO nanorods (**left**) and copper nanoparticles deposited onto ZnO nanorods (**right**).

Transmission Electron Microscopy was used to check the correct formation of ZnO nanorods, and to prove that copper nanoparticles were deposited onto nanorods surface (Figure 3) before loading the catalyst in the aerogel.

**Figure 4:**
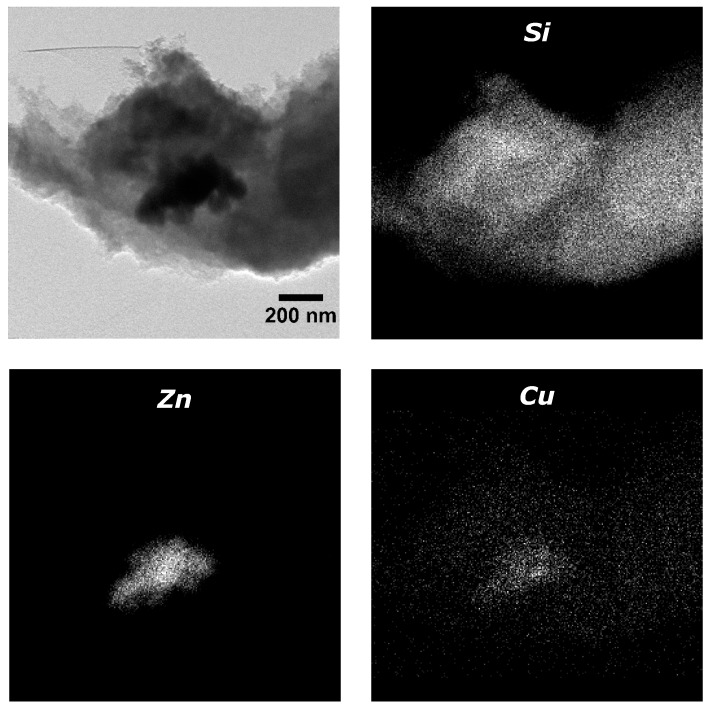
TEM image of a silica aerogel with catalyst and dark field to identify silica (Si), zinc (Zn) and copper (Cu) distribution.

These TEM images confirmed that ZnO nanorods were correctly created and discrete copper nanoparticles were formed on the ZnO surface without agglomeration as it was expected. Moreover, the impregnation of the catalyst inside the silica net was also studied in order to ensure catalyst was properly loaded (Figure 4). 

The distribution of silica, zinc and copper in the structure was obtained by using dark field technique selecting the reflections of each element. Silica was the main component and appeared throughout the structure, while zinc oxide nanoparticles were found all together inside the silica structure. Most of the copper nanoparticles were located in the zinc oxide area, indicating that they were correctly deposited onto ZnO nanorods. However, an important amount of copper nanoparticles were found away from zinc oxide nanorods given that they were not correctly deposited, affecting the efficiency of the process. This means that the Cu/ZnO catalyst nanostructure was affected during its introduction into the aerogel net. This also suggests the possibility of maldistribution of the catalyst inside the aerogel.

**Figure 5:**
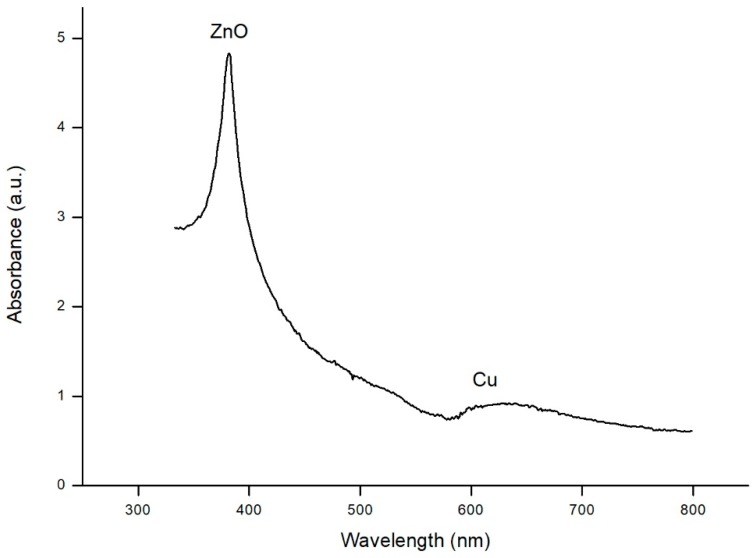
UV-Vis absorption spectra of ZnO/Cu catalyst.

Light absorption of this bimetallic catalyst was tested by UV-Vis Spectrophotometry (Figure 5) in order to prove both materials were absorbing light in the visible band (copper) and ultraviolet band (zinc oxide). ZnO nanorods exhibited a peak at 370 nm, and Cu nanoparticles induced a peak around 600 nm, which corresponded to the absorption of the range between orange and red colour [22]. The broad definition of a peak was due to the variable size of nanoparticles, moving in a narrow range of sizes but not enough to define a clear peak.

**Figure 6:**
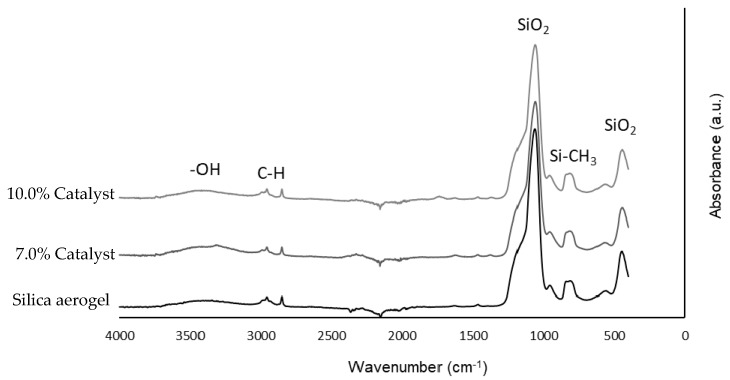
FTIR spectra of silica aerogel without and with catalyst load.

FTIR analysis was used to study the influence of catalyst loading on the structure of the aerogel. With this purpose, silica aerogel without catalyst was first analysed, in order to obtain the IR spectra of the silica material and identify all its chemical bonds. After that, two different amounts of catalyst load (7.0 and 10.0% in weight) were analysed to study if their addition affected the silica structure or the nanoparticles were located inside the porous structure without creating new bonds (Figure 6).

The main peaks of the silica structure were identified in the silica aerogel spectra. The most intense and broad peak was found at 1050 cm^−1^, with a shoulder at around 1200 cm^−1^ due to Si-O-Si asymmetric stretching vibrations [23]. The symmetric stretching vibrations of Si-O-Si were observed at 800 cm^−1^. When the catalyst was impregnated in the aerogel, no significant peaks appeared on the spectra, and the silica structure peaks were still correctly formed, concluding that catalyst loads did not affect the chemical structure of the aerogel.

**Figure 7:**
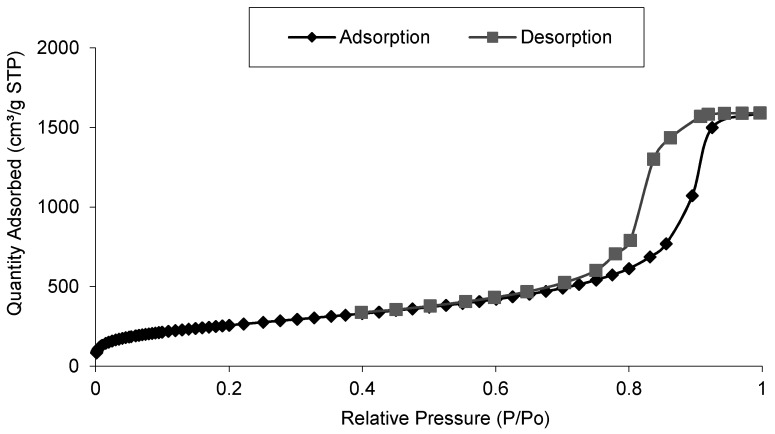
Isotherm adsorption—desorption curve for silica aerogels.

Same samples were analysed to determine the surface area and pore volume depending on catalyst load. Both properties were calculated from N_2_ isotherm adsorption—desorption curve, that showed for all the samples a type IV isotherm curve that is usual for mesoporous silica aerogels [24] (Figure 7). Almost no differences were found when 7.0% and 10.0% catalyst were loaded in the aerogel.

The BET surface area calculated was 845.3 m^2^/g for silica aerogels, and it was slightly reduced to 843.4 and 842.7 m^2^/g for 7.0 and 10.0% catalyst load, respectively. This indicated that the presence of catalyst did not have a big influence on the textural properties of the silica aerogel. However, the inclusion of the catalyst in the porous structure had an influence on the pore volume. For silica aerogels the BJH pore volume calculated was 2.91 cm^3^/g, being reduced to 2.46 and 2.29 cm^3^/g for 7 and 10% catalyst load, respectively. Loading more catalyst led to lower pore volume.

It is interesting that although the pore volume is reduced the surface area remains constant, in spite of the increase of catalytic material loaded. This effect could be owed to the already observed maldistribution (e.g., agglomeration) of the catalyst inside the aerogel structure which increases at higher loads. This could, in effect, leave most of the surface area free. This suggests that more studies are necessary for the improvement of the dispersion of the material and its characterization.

### 3.2. Carbon Dioxide Hydrogenation

After the proof of concept previously done working at 20 bar and 50 and 70 °C [18], a complete study of the influence of different parameters has been done. The variables whose influence has been studied were the preheating temperature of gases, the pressure, the gas inlet mass flows and the amount of catalyst. The results are presented as a function of the time elapsed since the flow (and illumination) was started.

#### 3.2.1. Influence of Temperature

The first parameter studied was the temperature for gases preheating. Visual light was the main source of energy for the carbon dioxide hydrogenation, although gases temperature could also have an influence on the process.

In order to analyze this variable, several experiments were performed. First of all, an initial experiment working without connecting the LEDs was carried out in order to analyze if the reaction could be performed only with temperature. Working always at 20 bar, 10.0% catalyst loaded and flows of 1 mL/min for hydrogen and 0.33 mL/min for carbon dioxide, experiments at 50, 100 and 150 °C were done and the conclusion was the same in all the cases: no conversion of carbon dioxide was produced.

**Figure 8:**
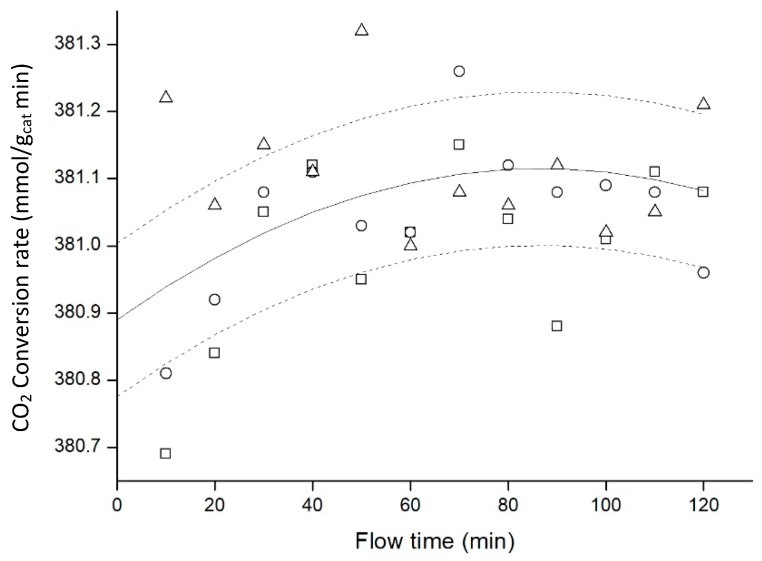
Influence of preheating temperature on the reaction.

After this, the same experiments were performed turning on the lights. In this case, carbon dioxide started to be transformed (Figure 8).

Figure 8 showed that preheating of gases had almost no influence on the CO_2_ conversion rate and the results were almost constant. This caused proximity between the data points, and thus error bars were excluded. However, the gases flow control system did not allow to avoid preheating. Flow controllers could not stabilize perfectly flows when the gases were cold, and they worked much better with a small heating. For this reason, it was decided to work always at 50 °C for the next experiments. 

#### 3.2.2. Influence of Catalyst Amount

**Figure 9:**
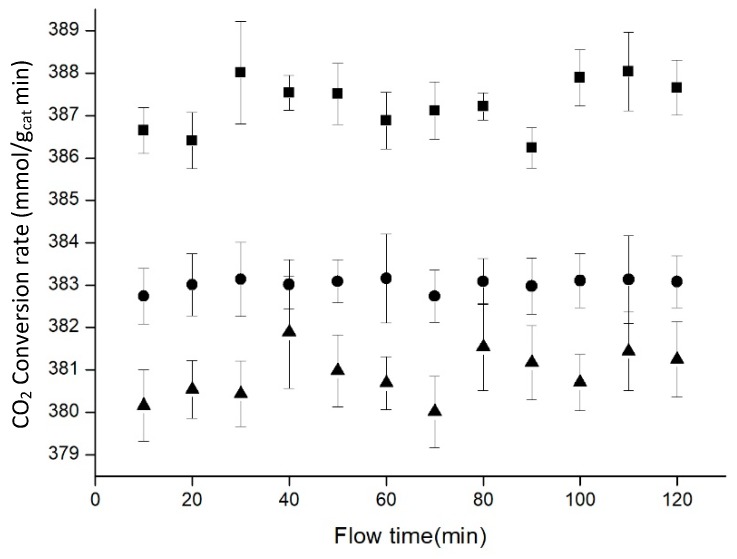
Influence of catalyst load on the reaction at 20 bar (7.0% ■, 10.0% •, 13.0% ▲). Data points represent the average of 3 experiments, with error bars corresponding to a 95% confidence interval.

The next variable studied was the amount of catalyst. Variation of the amount inside the aerogel was really easy, because the catalyst was directly diluted in the methanol needed as a precursor for the silica aerogel. However, 13.0% catalyst loaded in the aerogel was the maximum amount loaded, due to gelation problems for higher loads. These higher loads required longer gelation times, which promoted the precipitation of the catalyst, and as a result, the catalyst was not well distributed throughout the silica net. Because of this, experiments with 7.0, 10.0 and 13.0% catalyst deposited were performed, working always at 20 bar, 50 °C and flows of 1ml/min for hydrogen and 0.33 mL/min for carbon dioxide (Figure 9). 

Figure 9 showed that CO_2_ conversion rates were higher when the amount of catalyst decreased. Using more catalyst did not help to improve the process, attaining the opposite effect. Although metallic nanoparticles did not precipitate, they could be agglomerated inside the silica net. Agglomeration process entailed a reduction in the area used for carbon dioxide hydrogenation, and this could lead to a decrease in the CO_2_ conversion rate. In the same way, loading more catalyst reduced the light available generating a less transparent material and decreasing the volume of catalyst that was activated by light.

#### 3.2.3. Influence of Reactants Proportion

**Figure 10:**
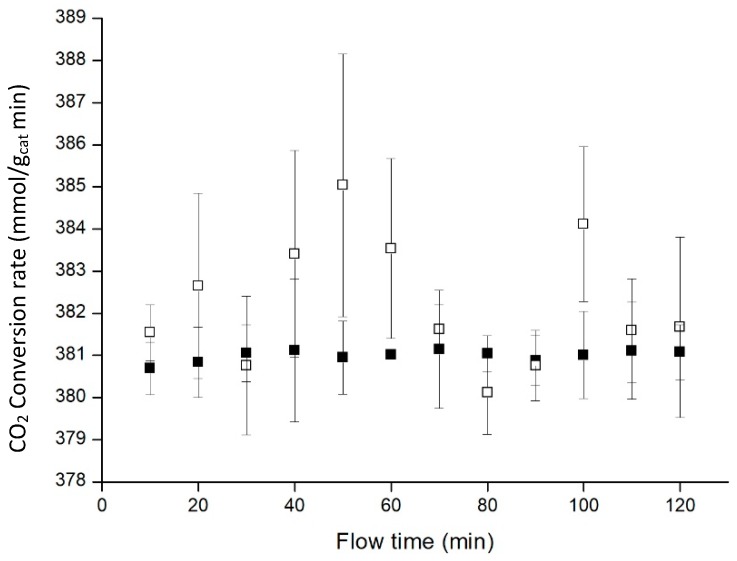
Influence of the reactants proportion (H_2_:CO_2_ 3:1 ■, 4:1 □). Data points represent the average of 3 experiments, with error bars corresponding to confidence interval 95%.

The influence of hydrogen and carbon dioxide flows on the conversion rate of CO_2_ was also analysed. They were mixed in a 3:1 proportion in order to test temperature and catalyst amount influence on the process, and it has been studied if adding more hydrogen could benefit the reaction (Figure 10). Pressure, temperature and catalyst load were 20 bar, 50 °C and 10.0% for all the experiences.

For these experiments, the CO_2_ flow was kept constant at the lowest controllable rate in our system (0.25 mL/min). Figure 10 indicated that increasing hydrogen proportion was not translated into a clear improvement for the process. In order to achieve 4:1 H_2_ to CO_2_ proportion, H_2_ flow remained constant at 1 mL/min, and CO_2_ flow was adjusted to 0.25 mL/min.

Nevertheless, the error involved at 4:1 H_2_:CO_2_ proportions is very large. This is related to the method that we have used to measure the CO_2_ conversion, which is based on gas chromatography, which is commonly used for larger flowrates (and therefore bigger samples). As consequence of the low flow rate, the signal corresponding to CO_2_ is diminished to a point where the changes in the measured concentration are very wide causing the results to oscillate more than in the 3:1 proportion. Thus, it is advisable to implement an in-situ method that complements the kinetic information obtained.

#### 3.2.4. Influence of Pressure

Finally, the last reaction variable whose influence has been studied was the pressure. Carbon dioxide hydrogenation is performed in the intersection between zinc oxide and copper nanoparticles [14], where at the same time carbon dioxide molecule was attacked and hydrogen molecule is retained enough time to produce the reaction [25]. Increasing the pressure of the system could help to this phenomenon and improve the process shifting the equilibrium in order to increase the concentration of the product.

In fact, for the case of methanol synthesis, both methanol selectivity and CO_2_ conversions have been proved to improve with higher pressures by Gaikwad, Bansode & Urakawa (2016) [26]. They demonstrated the strong influence of phase behavior and mass transfer and effective its control at higher pressures.

**Figure 11:**
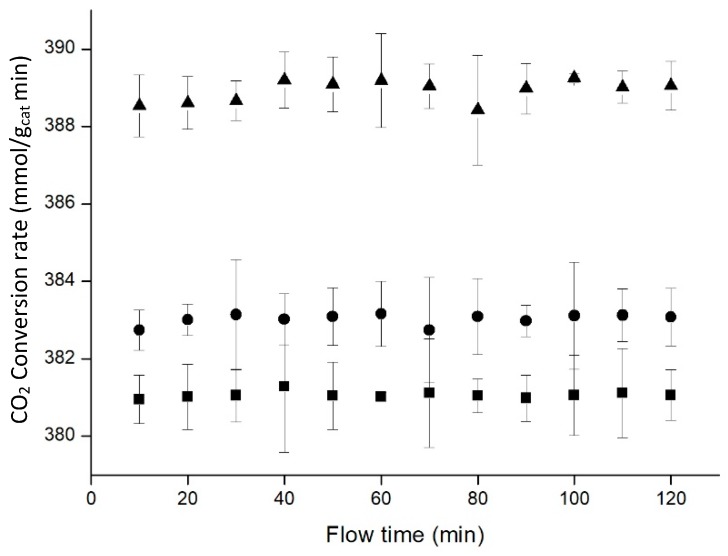
Influence of pressure on the reaction (20 bar ■, 30 bar •, 50 bar ▲). Data points represent the average of three experiments, with error bars corresponding to confidence interval 95%.

Initially, the pressure was varied between 20 and 50 bar to study its influence on the reaction (Figure 11). The experiences were performed working with 1 and 0.33 mL/min as hydrogen and carbon dioxide flows respectively, at a temperature of 50 °C and 10.0% of catalyst loaded in the aerogel.

Increasing the temperature improved the CO_2_ conversion rate, and this improvement was bigger for the highest pressure. The benefit obtained after increasing from 30 to 50 bar was much higher than increasing from 20 to 30 bar, which indicated that this improvement could continue increasing for higher pressures.

**Figure 12:**
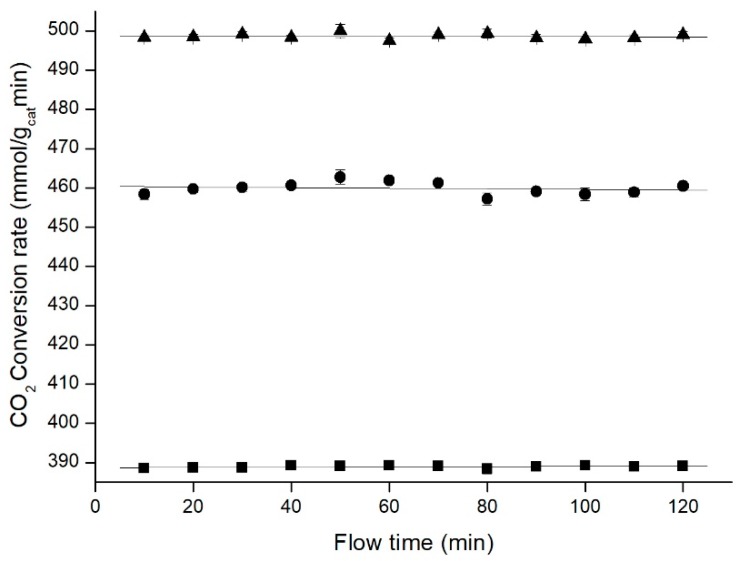
CO_2_ hydrogenation at high pressures (50 bar ■, 80 bar •, 100 bar ▲). Data points represent the average of 3 experiments, with error bars corresponding to confidence interval 95%.

With the aim of working at even higher pressures, a new carbon dioxide flow controller (Cori-Flow, Bronkhorst, The Netherlands) was acquired to enable work with CO_2_ also in supercritical conditions when pressure and temperature were above 73 bar and 31 °C. The introduction of this new flow controller allowed to increase the pressure up to 100 bar, and to study the reaction in these new conditions keeping all the other variables constant as in previous experiments (Figure 12).

These results continued showing an improvement in the CO_2_ conversion rate, particularly important after working in supercritical conditions for carbon dioxide. This large benefit continued until reaching 100 bar, which was the maximum pressure the experimental setup allowed to work. This is in accordance with Le Châtelier’s principle given that high pressures promote higher CO_2_ conversions, confirming the findings from Gaikwad, Bansode & Urakawa (2016) [26]. Moreover, it also shows the suitability of aerogels photocatalytic supports even under supercritical CO_2_ conditions.

## 4. Conclusions

Transparent silica aerogel was used as support for carbon dioxide hydrogenation with white light using a plasmonic catalyst composed of copper and zinc oxide. This nanocomposite has been impregnated in 0.5 ID glass microchannels with good adherence to be used to perform the reaction in the gas phase. The experimental setup built allowed precise control of the pressure, temperature and gases flow to study their influence on the reaction. The amount of catalyst loaded in the aerogel was also studied as a variable to improve the process.

Changes in temperature and gases flow did not affect CO_2_ conversion rates, not being useful to improve the process. An increase in the amount of catalyst was negative for the goal of improving the CO_2_ conversion rate, achieving better results for smaller amounts of catalyst. This could be owed to the maldistribution of the catalyst inside the aerogel at higher loads. The key parameter to enhance the results has been proved to be the pressure. Increasing pressure up to 50 bar improves CO_2_ conversion rate considerably, even though overpassing the critical point multiplied the benefit for the CO_2_ conversion rate. While other parameters provided a slight improvement, pressure improved up to 30% the CO_2_ conversion rate obtained. Aerogels are shown as attractive supports for photocatalytic gas phase reactions even under high-pressure conditions.
